# The effect of hydroalcoholic extract of *Ziziphora clinopodioides* L. on spatial memory and neuronal density of hippocampal CA1 region in rats with sporadic Alzheimer's disease

**Published:** 2019

**Authors:** Samaneh Sedighi, Maryam Tehranipour, Gholamhassan Vaezi, Vida Hojati, Hamid Hashemi-Moghaddam

**Affiliations:** 1 *Department of Biology, Damghan Branch, Islamic Azad University, Damghan, Iran.*; 2 *Department of Biology, Mashhad Branch, Islamic Azad University, Mashhad, Iran.*; 3 *Department of chemistry, Damghan Branch, Islamic Azad University, Damghan, Iran.*

**Keywords:** Alzheimer, Ziziphora clinopodioides Hippocampus, Spatial Memory

## Abstract

**Objective:**

Alzheimer's disease is a neurodegenerative disorder associated with gradual loss of cognitive and memory abilities. It was shown that the hippocampus is one of the first structures in the brain that is affected by the disease. *Ziziphora clinopodioides* (*Z. clinopodioides*) is a member of Lamiaceae family and contains various substances.

**Materials and Methods:**

In this experimental study, 72 adult male Wistar rats were used for behavioral and histopathologic studies. They were divided into nine groups included: control, negative control (Alzheimer), positive control (Alzheimer's treated with rivastigmine), aCSF (artificial cerebrospinal fluid) + *ziziphora* extract with doses of 200,400, and 600 mg/kg, and STZ (stereptozotocine)+*ziziphora* extract in 200,400,600 mg/kg doses. The injury was created with bilaterally intraventricular injection. The spatial memory was studied by passive avoidance test and neuronal density was evaluated by dissector method. To examine the histopathological lesions, Congo red and toluidine blue staining were done. Data were analyzed by ANOVA Minitab software.

**Results:**

The memory index (neuronal density and passive avoidance test results) showed a significant decrease in negative control group compared to control (p≤0.001). Treatment with the hydroalcoholic extract at the doses of 400 and 600 mg/kg showed a significant increase in memory index in rats with Alzheimer's disease (p≤0.001). The effect of 200 mg/kg extract was not significantly different from that of the negative control group. The results of histological analysis indicated beta-amyloid plaques formation in the control group as compared to the negative control group while treatment with the extract at the doses of 400 and 600 mg/kg, significantly reduced beta-amyloid plaques formation.

**Conclusion:**

These findings suggest that the extract of *Z. clinopodioides* can improve Alzheimer's condition and alleviate memory and histopathologic damages; also, it decreases beta-amyloid plaques and apoptosis in CA1 region of the hippocampus.

## Introduction

Alzheimer’s disease (AD) is the major cause of dementia, which is characterized by excessive loss of memory and learning ability, and other cognitive functions of the brain (Kroner, 2009 ▶). The mechanism(s) underlying cognitive and memory impairment in AD were not identified accurately; but, it is known that the hippocampus is one of the earliest brain structures damaged in this disease (Janson et al., 2004 ▶). The hippocampus is part of the limbic system located in middle temporal lobe and plays important roles in learning and retrieving information from short-term to long-term memories (Herring et al., 2009 ▶). 

Intracerebroventricular injection (i.c.v) of streptozotocin (STZ) is one of the suitable approaches for inducing AD in experimental animals (Chen et al., 2013 ▶; Rowan et al., 2007 ▶). AD caused in this model is known as Sporadic Alzheimer's Disease (SAD) which is seen in >90% of humans with AD (Hoyer et al., 2000 ▶; Lannert and Hoyer, 1998 ▶). Also histopathological studies showed that i.c.v injections of STZ cause changes in brain tissue, especially in hippocampal region, which is similar to the tissue changes related to SAD (Agrawal et al., 2009 ▶). Despite the advances in treatment and management of AD, it is still challenging and no definitive treatment has been found for it. Furthermore, the drugs used against AD, such as rivastigmine, have their advantages and disadvantages. Rivastigmine, which was first introduced in Switzerland in 1997, was approved for AD treatment (Cummings and Winblad, 2007 ▶; Yang and Keating, 2007 ▶).

Many compounds extracted from plants were shown to be effective in improving memory, and preventing and also treating AD and related mental disorders (Hajiaghaee and Akhondzadeh, 2012 ▶; Xiao and Tundis, 2013 ▶). Lesser side effects, better therapeutic response and lower treatment costs are the most important features which make the medicinal plants and their derivatives attractive to be considered for AD treatment (Williams and Spencer, 2012 ▶). One of the treatment approaches against AD is to use antioxidant compounds. These compounds can prevent the progression of the disease by removing free radicals (Butterfield et al., 2001 ▶).

*Ziziphora clinopodioides *(*Z. clinopodioides*) is a medicinal plant, known as “*Ziziphora”*, belonging to the Lamiaceae family, and its aerial parts are consumed as a spice. This plant is found in most parts of north and west, and also some parts of center of Iran with nine endemic subspecies (Squire and Zola, 1996 ▶). All parts of this plant are used in herbal medicine. 

*Z. clinopodioides* plays effective and sedative roles in treatment of gastrointestinal diseases with psychological origin, and also diarrhea with nervous stimulation. This plant also has an effect on gastrointestinal and liver regulation, in particular nutrition digestion (Gill et al., 2009 ▶). Limited researches was done on *Ziziphora* and no study evaluated its effect on the neurological system. The aim of this study was to investigate the protective role of *Ziziphora* extract against SAD induced by i.c.v injection of STZ in cerebellum hemispheres of male Wistar rats and the results were compared with those obtained following rivastigmine administration.

## Materials and Methods

**Animals**

In this study, 72 male Wistar rats, weighting 230-280g, were prepared from Pasteur institute, Tehran, Iran. Each four animals were kept in a standard cage with unlimited supply with food and water. The temperature of animal storage room was set at 24°C with 12-hr/ 12-hr light/dark regular cycles. Animals’ experiments were done in accordance with the recommendations of Animal Nutrition Support Laws (NIH). Animals were divided into the following nine groups (n=8 in each):

1) Control

2) STZ (Negative control): surgery was performed and i.c.v injection of STZ solution in synthetic cerebrospinal fluid (CSF), and saline i.p were carried out.

3) STZ+Rio (Positive control): surgery was performed and i.c.v injection of STZ (aCSF) solution on the first and third days, and i.p injections of rivastigmine solution in saline at 0.3mg/kg/day (Mahdy et al., 2012 ▶; Zhou et al., 2016 ▶) were carried out for 21 days.

4-6) aCSF+ziziphora: surgery was performed and i.c.v injection of artificial cerebrospinal fluid (aCSF) on the first and third days, and i.p injections of the extract of *Ziziphora* at three doses of 200, 400 and 600 mg/kg/day in saline solution were performed for 21 days.

7-9) STZ+ziziphora: surgery was performed and i.c.v injections of STZ (aCSF) on the first and third days, and i.p injections of *Ziziphora* extract at three doses of 200, 400 and 600mg/kg/day in saline solution were done for 21 days.

**Extraction**

Ziziphora was collected from the Binaloud mountain located 3 km away from the Soqand Village, around Neishabour, Khorasan Razavi, Iran. The plant identity was confirmed in the botanical lab of Mashhad Azad University with herbarium No.10313. Dried leaves of Ziziphora were completely milled for each turn in the same day and they were extracted by Soxhlet hydroalcoholic method (Kyari, 2008 ▶). 

**Surgery**

For i.c.v infusion of STZ (S0130, Sigma), two guide cannulae were surgically implanted inside the lateral ventricles of brain under stereotaxic surgery. Initially, the animals were anesthetized by i.p injection of 60 mg/kg bodyweight of ketamine (Alfasan, Woerden, Holland), and xylazine (Alfasan, Woerden, Holland), 20 mg/kg bodyweight (Naghizadeh et al., 2013 ▶). Then, the hair of animals’ head was shaved and the animals were placed in stereotaxic (Stoelting Co., USA). Shaved scalp was cleaned and disinfected using alcohol. An interior-posterior gap was created with an approximate length of 2.5cm in the middle of animals’ head (from both sides of eye to the back of the ear). Subcutaneous tissue was removed and the area was completely cleaned to determine the locations of Bregma and Lambda.

Using Palazzi and Bordier rat brain atlas (Palazzi and Bordier, 2008 ▶), as well as a pilot sample of the cannula location of lateral ventricles of brain with a coordinate of 0.8 mm to the backside from the Bregma, cannulation was performed in the brain, 1.5 mm to the sides and 3.6 mm from its surface (Paxinos and Watson, 1986 ▶).

After cannulation, and during the recovery (7 days), the rats with normal weight and activity, received 3 mg/kg of STZ (Sigma-Aldrich, St. Louis, MO, USA) diluted in an artificial cerebrospinal fluid (aCSF). It should be noted that aCSF included NaCl 147 mM; KCl 2.9 mM; MgCl_2_ 1.6 mM; CaCl_2_ 1.7 mM; C_6_H_12_O_6_ 2.2 mM with pH 7.4 (Agrawal et al., 2009 ▶) and it was injected using a Hamilton syringe for 10 min on days 1 and 3 on each side of lateral ventricles of the brain (Mehla et al., 2013 ▶). The injection volume was 5 μl for each side.

**Passive avoidance test**

The shuttle box is used to test the active and passive avoidance for evaluation of the memory. Inactive avoidance behavior test was performed on day 21 after the first injection of extract using the shuttle box (BPT Co., Tehran, Iran). Animals were acquainted to the device during the adaptation phase on the first day for 5 min, and by opening the valve between the two boxes, they were allowed to move freely from the bright side to the dark side.

On the day after the adaptation stage, the animals were placed in bright side of the device while the valve between the two boxes was closed. Before entering the bright side, the hands and feet of the animal were moisturized with saline to improve feeling of the electric shock. Time spent in the bright side was 30 sec. After that, the valve was opened, rat went to the dark and the time duration was recorded; if the spent time exceeded 120 sec, the rat was removed from the experiment. After entering the dark side, the valve was closed and after 5 sec, and a 1.5 mA electric shock was given to the animal for 2 sec (Miu et al., 2003 ▶; Moazedi et al., 2015 ▶). After 20 sec, the rat was removed from the device, and another animal was trained in the same way. The time taken for each animal to move from the bright box to the dark one was considered the latency of entering to dark box (Initial latency=IL) (before the shock), which was used to control the visual and motor ability. 

At the test stage, 24 hr after the previous stage, the animals were evaluated. Trained rats were placed in the bright box for 30 sec and then the valve between the two boxes was opened, and the time spent for entering the dark box, was measured. This time, the step through latency=STL, should be lasted up to 600 seconds long, and if the rat did not enter the dark side, another 600 sec was given (Miu et al., 2003 ▶; Moazedi et al., 2015 ▶).

**Histological examinations**

Following the behavioral test, deep anesthesia was induced and perfusion was performed. Then, animals’ brain was removed for histological studies. 10% formaldehyde solution used for fixation was administered through the main vessels; the brain was removed and put in a similar solution for final fixation. The tissues were prepared by histochemical and tissue passage and sectioned serially at the thickness of 7 µm by a microtome (Leica RW2255 Germany). For staining, samples were stained with toluidine blue and evaluated for the neuronal density by dissector method, and the Congo staining with red color was used to examine the beta-amyloid plaques in hippocampus. Congo red dye forms nonpolar hydrogen bonds with amyloid and red to apple green birefringence occurs when viewed by polarized light due to alignment of dye molecules on the linearly arranged amyloid fibrils. A high pH enhances the non-polar hydrogen bonding of Congo red and amyloid (Nilsson 2004 ▶).

The CA1 region in hippocampus was photographed by a Nikon microscope (Japan) (West, 1999) and neural density was examined by dissector method.

The dissector method counts the neurons in a reference frame. If the neuron is in both boxes, it is not counted, but if the neuron is located inside the reference frame but not in the next frame, it is counted. After neuron counting, neural density was calculated based on following formula:

ND=ΣQ/Σframe×V dissectorND: neural density

ΣQ: total counted neurons in one sample

Σframe: Total sampling times in one sample

V disecector: Volume of sampling frame

V dissector=A frame×H

A frame: The area of the sampling frame

H: distance between two subsequent sections or the thickness of each section

**Statistical Analysis**

Data were analyzed by, ANOVA, and t-test (for binary comparison of groups) using Minitab v.16. A p≤0.05 was considered significant.

## Results

**Passive avoidance test**

**Figure 1:**
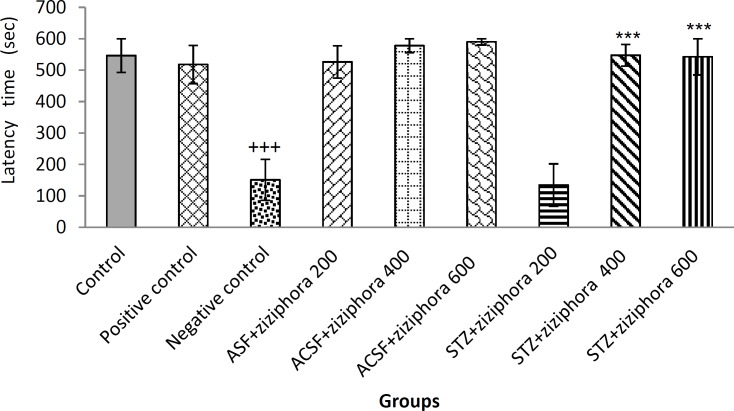
Effect of hydroalcoholic extract of Ziziphora given for 21 days on memory test and passive avoidance learning in control, positive control, negative control, and aCSF+*ziziphora* groups as well as groups treated with *Ziziphora* extract (n=8). The data are presented as mean±SEM.

Single trail passive avoidance behavior test is an inactive avoidance test and delayed response beginning. In this test, the average of delay time in entering the dark room was compared among the groups. The results showed that STZ injection in the lateral ventricles could negatively affect the process of stabilizing learning. The comparison of mean of delay time among aCSF+ziziphora, the control and negative control (Alzheimer's group) groups indicated a significant difference between the control and negative control groups. There was a significant decrease in mean delay time in negative control group compared to control group (p≤0.001), while the mean delay time for entering was not significantly different between the aCSF+ *ziziphora* and control groups (Figure 1).

Rats treated with STZ+ziziphora 400 and 600 showed a significant increase in mean delay time compared to negative control group (p≤0.001), but did not show significant difference compared to control group. However, comparison of delay time did not show significant differences between STZ+ziziphora 400 and 600 and the positive control group (Figure 1).

All these findings indicated that the extract at the dose of 200 mg/kg did not improve the disorder while groups treated with ziziphora 400 and 600 mg/kg were improved to levels comparable to rivastigmine as an effective medication for Alzheimer's disease. Also, there was no significant difference in delay time between a CSF+ziziphora and control groups (Figure 1).

**Neuronal density**

Mean neuronal density of the CA1hippocampal region as 3786±502 in the control group. In negative control group, neuronal density was 2221±117 which was significantly lower than the control group (p≤0.001), indicating accurate induction of Alzheimer's disease in negative control group. In rats of STZ+ziziphora 200mg/kg group, neuronal density was 2231±109 which was significantly lower than the control group.

**Figure 2:**
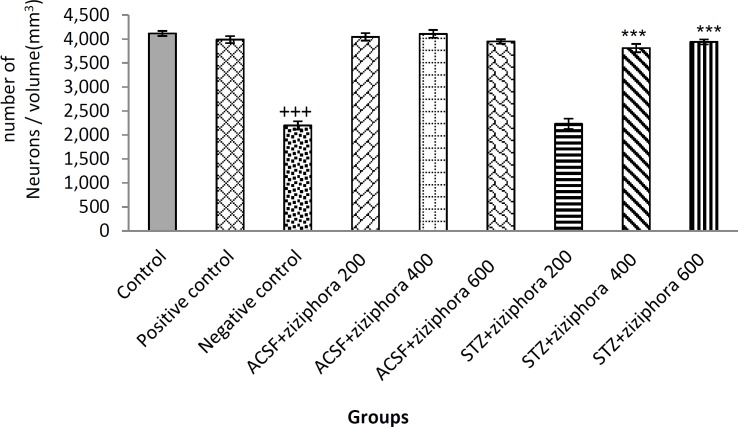
Effect of hydroalcoholic extract of *Ziziphora* given for 21 days on neuronal density in CA1 region of hippocampus in control, positive control, negative control, and aCSF+*Ziziphora* groups as well as groups treated with* Ziziphora* extract (n=8). The data are presented as mean±SEM.

However, in STZ+ziziphora 400 mg/kg and STZ+ziziphora 600 mg/kg groups, neuronal density was 3594±192 and 1834136, respectively. Moreover, in aCSF+ziziphora 200 mg/kg and aCSF+ziziphora 400 mg/kg, neuronal density was 4044±78 and 4058±115, respectively. No significantly difference was found between the control group and treatment and sham groups in terms of neuronal density. However, there was a significant difference between control group and aCSF+ziziphora 600 mg/kg group (p=0.032) (Figure 2).

 Meanwhile, comparison of neuronal density between the positive control group and the STZ+ziziphora groups showed significant reduce with dose of 200mg/kg, (P≤0.001). However, there was no significant difference between aCSF+ziziphora 400 mg/kg and 600mg/kg groups (Figure 2).

*** Significant difference between treatment groups and negative control group (p≤0.001).

+++ Significant difference between control group and negative control group (p≤0.001).

*** Significant difference between treatment group and negative control group (p≤0.001).

+++ Significant difference between control group and negative control group (p≤0.001).

**Histology findings**

Sections stained by Congo red method, were examined for the formation of beta-amyloid plaques in hippocampus, the animals in Alzheimer’s disease group received different doses of extract for a period of 21 consecutive days to evaluate the effect of extract on removing the beta- amyloid plaques.

**Figure 3:**
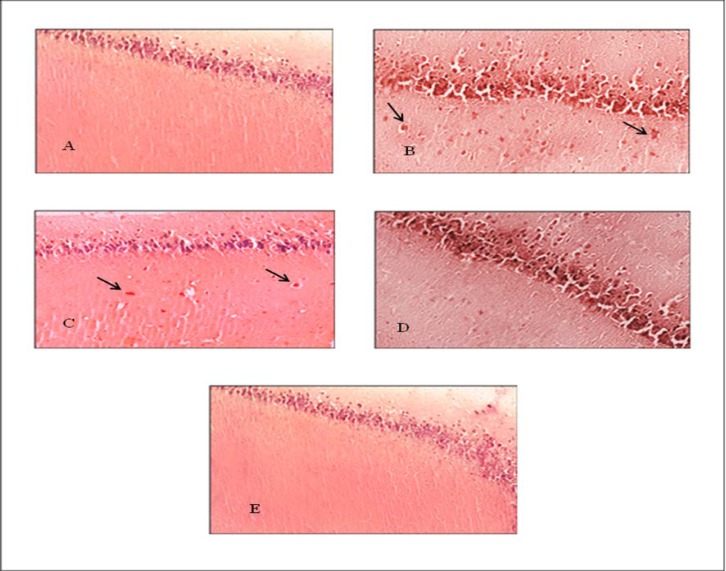
. A. The hippocampus in control group with no amyloid plaque.

Cerebral sections obtained from the group treated with *Ziziphora* extract 200mg/kg were comparable to those of the negative control group samples, which did not receive the extract. The plaques were clearly visible, indicating that *Ziziphora* extract 200mg/kg had no effect on plaque cleansing. However, extract 400mg/kg given for a period of 21 consecutive days reduced the number of hippocampal plaques. However, comparison negative control and 200mg/kg dose groups did not show significant changes in number of plaque and density. Extract 600mg/kg caused more plaques cleaningin hippocampal region, so that the samples of this group were very similar to normal group. This showed that the hydroalcoholic extract of *Ziziphora* dose-dependently reduced beta-amyloid plaques formation in the brain of the animal with AD (Figure 3).

**Figure 4:**
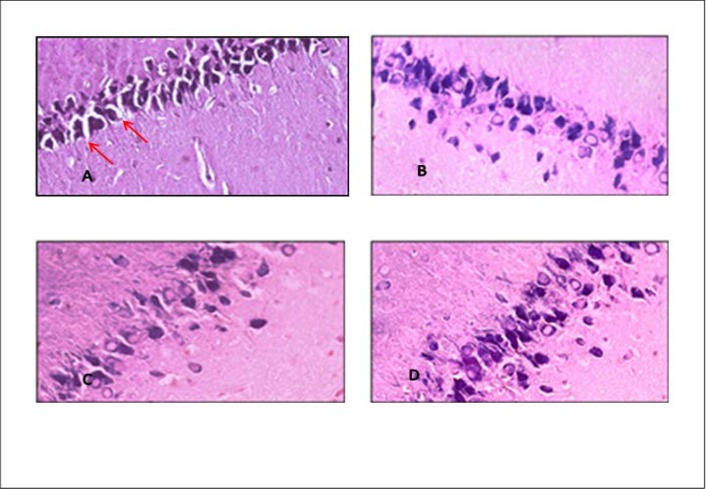
Cross sections of the CA1 region in hippocampus stained with blue toluidine. In the negative control group, aggregated and degenerate neurons were observed, so that intercellular space was also increased. (A) compared to normal neurons in control group (B) injury rate is significantly reduced in groups treated with *Ziziphora* extract. In *Ziziphora* extract 400 (C) and 600mg/kg (D), the degeneration and intercellular spaces of the cells have been reduced. 40X magnification

The histopathologic study of CA1 of hippocampal region sections, which were stained with toluidine blue, showed that normal neurons and other glial cells in control and sham groups, with round and distinct nucleus, and normal cytoplasm is without any damage (Figure 4). The analysis of sections prepared from the negative control group indicated shrinkage and dense nucleuses representing the pyknotic and necrotic neurons. In groups treated 400 and 600 mg/kg of* Ziziphora*, improvement of CA1 region were clearly seen.

B. The hippocampus in negative control group 21 days after streptozotocin injection indicating the presence of beta-amyloid plaques appeared as red spots.

C. The hippocampus of animals treated for 21 days with 200mg of *Ziziphora* extract. Beta-amyloid plaques are visible; no difference was observed between 200mg of *Ziziphora* extract and the control group.

D. The hippocampus of animals treated with 400mg of *Ziziphora* extract. Beta-amyloid plaques were decreased compared to negative control group.

E. The hippocampus of animals treated with 600mg of *Ziziphora* extract. Number and density of beta-amyloid plaques were markedly decreased so that no difference was observed between this group and the control group.

## Discussion

It was shown that i.c.v injection of STZ is one of the suitable models to induce and study AD in laboratory animals (Chen et al., 2013 ▶; Rowan et al., 2007 ▶). This substance changes the activity of microglia and disrupts myelination through induction of oxidative stress and neuronal inflammation in regions involved in cognitive processes related to memory (Tota et al., 2011 ▶). Histopathological studies also showed that STZ i.c.v injections cause brain tissue changes, degeneration and pyknosis reactions, especially in the hippocampus, and accumulation of the beta amyloid peptides (Salkovic‐Petrisic et al., 2006 ▶), which is similar to the histopathologic changes of SAD (Agrawal et al., 2009 ▶; Biasibetti et al., 2017 ▶; Singh et al., 2013 ▶). Cognitive impairment in AD may occur due to synaptic disorders caused by accumulation of beta-oligomeric amyloid before neuronal degeneration (Klyubin et al., 2005 ▶). The pathway of perforant (PP) from the entorhinal cortex to the hippocampus is susceptible to the initial accumulation of beta-amyloid peptide in the brain in AD (Rowan et al., 2007 ▶). Studies have shown that administration of aggregated beta-amyloid plaques (Aβ) makes changes in long term potentional (LTP) in the hippocampus and consequently leads to cognitive dysfunction and impairment of spatial of learning and memory in rodents (Babri et al., 2012 ▶; Lannert and Hoyer, 1998 ▶). In the present study, i.c.v injection of STZ caused neuronal damage, decreased the number of neurons and resulted in accumulation of beta-amyloid plaques. Using the extract of *Ziziphora* at high doses (400 and 600mg/kg) significantly increased neuronal density (Figure 2). *Ziziphora* includes various substances including flavones, flavonoids, oxygenated monoterpenes, etc. The flavones, and flavonoids are considered highly effective antioxidants (Aliev et al., 2008 ▶; Rice-Evans et al., 1996 ▶). Phenolic agents show antioxidant properties. Studies have shown that phenols increase neuronal expansion and induce neuroprotection (Kulišić et al., 2007 ▶) and antioxidant agents can prevent nerve cell death by reducing the oxidative stress caused by beta-amyloid peptide (Aliev et al., 2008 ▶).

*Ziziphora* contains phenolic compounds such as geraniol, carvacrol, thymol and alpha-pinene (Kim et al., 1996 ▶; Sul et al., 2009 ▶). The essential oil of* Ziziphora* can inhibit free radicals due to the presence of certain compounds such as thymol and carvacrol. The antioxidant activity of thymol and carvacrol is because of the presence of phenolic groups that act during lipid oxidation as hydrogen-donor agents to peroxide products and thus delay the formation of hydroxyperoxide (Dehghan et al., 2010 ▶). Therefore, some of the beneficial effects of *Ziziphora* observed in the present study can be attributed to the presence of phenolic compounds, specially thymol and carvacrol, as these compounds reduce lipid peroxidation and oxidative stress, and protect and expand neurons; current findings are consistent with previous studies about beneficial effects of *Ziziphora* (Salkovic‐Petrisic et al., 2006 ▶). 

The present study showed that learning and memory in male Wistar rats are significantly reduced by STZ injection and Alzheimer's induction. STZ causes impairment in learning and memory capacity by inhibition of insulin receptors in the brain through reduced glucose consumption, decreased activity of glycolytic enzymes (Reisi et al., 2009 ▶), decreased energy production (Salkovic‐Petrisic et al., 2006 ▶), reduced activity of choline acetyltransferase enzyme and cholinergic afferent neuronal damage (Li et al., 2008 ▶), as well as decreased activity of the catechol-aminergic system (Grünblatt et al., 2007 ▶). 

*Ziziphora* extract enhanced the memory function in rats in the passive avoidance learning test (Figure 1). Also, the cholinergic system is responsible for storing and retrieving memory, and its supression is associated with increased memory damage; so it seems that increased levels of acetylcholine improve the symptoms of memory impairment in AD (Jain et al., 2001 ▶). Also, in AD, cholinergic neurons in basal forebrain area disappear gradually, resulting in memory impairment in these patients (Nezhadali and Shirvan, 2010 ▶).

Monoterpenes inhibit the acetylcholinesterase enzymes (Lane et al., 2004 ▶), leading to increased acetylcholine level which bind to both nicotinic and muscarinic receptors, and as a result, cholinergic system in perceptual processes is regulated (Carreiras and Marco, 2004 ▶; Perry et al., 1998 ▶). Monoterpenes, carvacrol and thymol are the main components of *Ziziphora* extract (Kim et al., 1996 ▶; Sul et al., 2009 ▶). The inhibitory effect of carvacrol on acetylcholine esterase is ten times stronger than thymol (Kennedy et al., 2004 ▶). For this reason, approved drugs that are used to control the disease, are cholinesterase-inhibiting drugs (Xing et al., 2010 ▶) such as rivastigmine (Cummings and Winblad, 2007 ▶; Kazmierski et al., 2018 ▶; Yang and Keating, 2007 ▶). In this research, *Ziziphora* extract improved the memory performance by affecting hippocampal cholinergic branches and cholinesterase enzyme inhibitors. In this study, the groups treated with *Ziziphora* extract 400 and 600mg/kg doses did not show significant differences compared to positive control group (i.e. the group treated with rivastigmine), and this indicates the ability of *Ziziphora* extract to compete with rivastigmine.

So far, no research was performed on effects of *Ziziphora* on memory or the nervous system. At the same time, these findings are consistent with earlier studies on the effects of other members of the *Lamiaceae* family such as thyme, rosemary, and dracocephalum on memory improvement (Perry et al., 2018 ▶; Perry et al., 2000 ▶; Rabiei et al., 2015 ▶).

Finally, the present study showed that treatment with *Ziziphora* extract significantly prevented memory damages caused by STZ i.c.v injections in the brain. This result demonstrated the potential ability of *Ziziphora* extract in prevention of Alzheimer’s associated neurodegenerative disorders and cognitive problems; however, to examine other effects of *Ziziphora* extract and their possible mechanisms, more paraclinical studies are needed.
